# Basosquamous Cell Carcinoma of the Nipple-Areola Complex—Report of a Case

**DOI:** 10.3390/medicina59020316

**Published:** 2023-02-08

**Authors:** Gabriele Raimondo, Gaetano Gallo, Giuliano D’Onghia, Giovanni Gabriele, Luciano Izzo, Andrea Polistena, Luca Esposito, Paola Giancontieri, Leonardo Macci, Vito D’Andrea, Enrico Fiori, Luigi Basso

**Affiliations:** 1 Department of Surgery, Policlinico “Umberto I”, “Sapienza” University of Rome, Viale del Policlinico 155, 00161 Roma, Italy; 2 Department of Radiological, Oncological and Anatomo-Pathological Sciences, Policlinico “Umberto I”, “Sapienza” University of Rome, Viale del Policlinico 155, 00161 Rome, Italy

**Keywords:** basosquamous cell carcinoma, squamous cell carcinoma, nipple areola complex, non-melanoma skin cancers, mastectomy, sentinel lymph node biopsy

## Abstract

Basosquamous cell carcinoma (BSCC) is a rare malignancy usually arising on sun-exposed areas of the skin. BSCC is described as a rare variant of Basal cell carcinoma (BCC) which shows clinical and microscopic features of both BCC and of Squamous cell carcinoma (SCC). We report the case of a 70-year-old male with a cutaneous lesion of the nipple-areola complex (NAC); to the best of our knowledge, this is the first ever reported patient with BSCC in this area. The lesion had a fast growth, but, due to the COVID19 crisis, the patient only came to our observation one year after onset of this condition. Physical examination showed a bleeding red ulcerated lesion that involved the NAC, measuring 27 mm × 20 mm. Biopsy showed a BSCC. Pre-operative breast ultrasound scan, mammogram and MRI were all performed before surgery, which consisted of simple mastectomy and sentinel lymph-node biopsy. The patient was discharged home on the 4th post-operative day, and at 18-month follow-up there are no signs or clinical evidence of local recurrence or metastases. Diagnosis of BSCC of the nipple-areola complex requires high index of suspicion and a thorough differential diagnosis, management, and suitable radical treatment due to well described high rates of recurrence and of metastases. Differential diagnosis with similar lesions (e.g., Paget’s disease, Bowen’s disease, BCC, and SCC) should also be taken into account.

## 1. Introduction

Basal Cell Carcinoma (BCC) and Squamous Cell Carcinoma (SCC) are the most frequent types of Non-Melanoma Skin Cancers (NMSC) [1]. Basosquamous Cell Carcinoma (BSCC) is described as a rare variant of BCC which shows clinical and microscopic features of both BCC and of SCC [2,3]. Several authors [4] consider Metatypical Basal Cell Carcinoma (MBCC) a synonym for BSCC. Cutaneous ultraviolet (UV) exposure is closely related to the development of skin cancer, which, however, is of multifactorial origin. In relation to UV exposure alone, there are many studies that prove its role in the development of skin cancer [5,6]. While the sun is the most common source of cutaneous UV exposure, many questions remain unanswered regarding the exact mechanism causing skin cancer following UV exposure. The p53 suppressor gene, which is frequently mutated in skin cancers, seems an early target of UV radiation induced neoplasms [7]. Hence, BSCC, as well as BCC and SCC, mainly arises in sun-exposed areas of the body, such as head and neck [2,8].

We report the case of a 70-year-old male with a cutaneous lesion of the nipple-areola complex (NAC). Biopsy showed BSCC, and a simple mastectomy and sentinel lymph-node biopsy were performed. By “simple mastectomy” we indicate removal of the mammary gland, of the NAC, and of the pectoralis major fascia. The patient was free from recurrence and metastases at 18 month follow-up. To the best of our knowledge, this is the first case of BSCC arising in the unexposed NAC.

## 2. Case Presentation and Results

**Figure 1:**
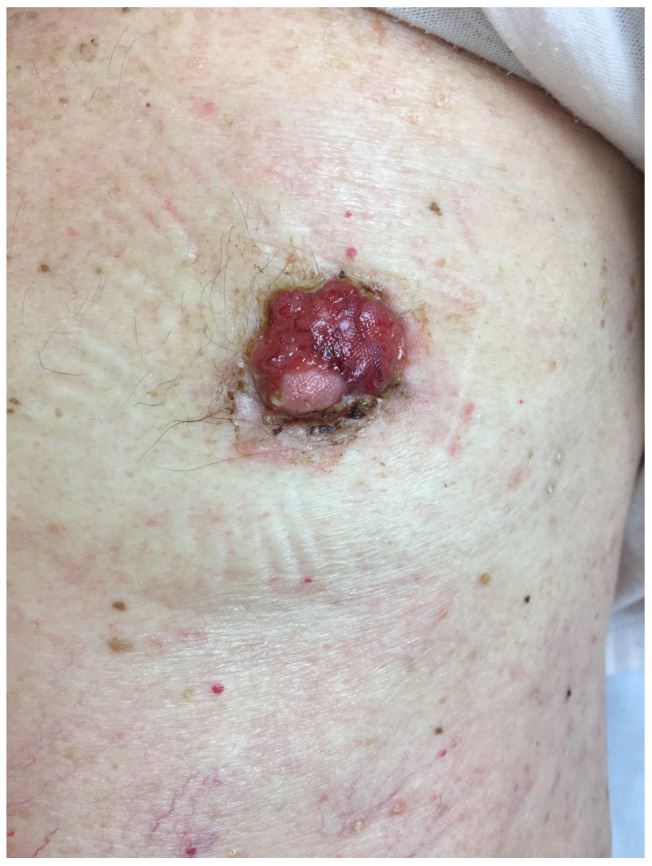
Enlarged left nipple-areola complex. Clinical appearance of BSCC on the nipple. Erosion on the surface represents the biopsy site. Dermoscopy showed absence of a pigment network and the presence of arborizing vessels, large blue-gray ovoid nests, multiple blue-gray globules, leaf-like areas, spoke wheel areas, and ulceration.

The present case report was developed according to the CARE checklist [9] (Supplementary Materials). A 70-year-old man, retired bank employee without any personal or family history of malignancy or of extreme sun exposure, presented with a plaque tumor on the left NAC. The lesion had a rapid growth, but, due to the COVID19 crisis, the patient only came to our observation one year after its onset. Physical examination showed a bleeding, red, ulcerated lesion that involved the NAC, measuring 27 mm × 20 mm. The surrounding skin was erythematous and edematous (Figure 1). No evidence of axillary lymphadenopathy was clinically observed.

**Figure 2:**
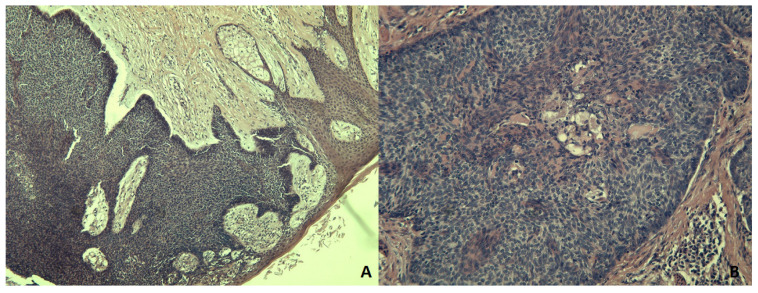
BSCC. (**A**): Islands of basaloid cells with peripheral palisading, limit with the overlying epidermis (HEX50) (**B**): Evidence of squamous differentiation in the basaloid island in the center of this image (HEX100).

Breast ultrasound scan, mammogram, and MRI were all performed to rule out Paget’s Disease, and these tests excluded involvement of mammary gland and of axillary lymph nodes. BCC or SCC were considered for clinical differential diagnosis; therefore a punch biopsy was taken from the lesion. Microscopy showed histological features of BSCC. Consequently, a simple mastectomy with sentinel node biopsy were performed. The pathology report revealed that the surgical specimen consisted of mammary gland measuring 10 cm × 5 cm–5 cm × 3.5 cm, covered by a skin lozenge of 10 cm × 5.5 cm, harboring areola and nipple. Both the areola and, to a lesser degree, the nipple showed an ulcerative lesion with a maximum diameter of 2.5 cm. On the cut section, the lesion extended to the underlying dermis, reaching, in some areas, the edge of the subcutaneous tissue. At microscopic examination, the lesion showed a typical architecture of islands and trabeculae, mostly composed of cells with dark nuclei, and scant cytoplasm, with several areas with wider cells and eosinophilic cytoplasm, occasionally around and near extracellular acidophilic material, evocative of squamous cells differentiation (Figure 2). Immunohistochemical profile of the lesion included positive stain for low molecular weight keratin, CD10 and BerEP4 in basal cells, intermingled with positive stain for high molecular weight keratin and epithelial membrane antigen, in squamous-like cells. The final diagnosis was of BSCC of areola and nipple. Sentinel node biopsy was negative for malignancy, and no further treatment was required, as there were no signs of residual disease.

The patient was discharged home on the fourth post-operative day. Following surgery, no medical treatment was employed as at microscopic evaluation clear margins of resection resulted. At the 18 month follow-up the patient is doing well, without clinical evidence of local recurrence or metastases. 

## 3. Discussion

BSCC, also named Metatypical Basal Cell Carcinoma or MBCC, is a rare skin cancer with an incidence of 1.2–2.7% of all NMSC [10,11]. BSCC shows pathological features of both BCC and SCC, with a non-specific clinical presentation. Several disputes exist on classification and pathogenesis of BSCC. Since 2005, the World Health Organization (WHO) defined BSCC as a “tumor with infiltrative growth, with areas of keratinization and/or formation of intracellular bridge, in the setting of prototypic proliferative stromal reaction” [3]. Garcia et al. [2] proposed to define BSCC as “an infiltrative growth subtype of BCC that shows areas of BCC and SCC with or without a transition zone and a fibroblast, rich collagenized stroma”.

Standard therapeutic guidelines for the treatment of BSCC have not been issued, and wide local excision, Mohs micrographic surgery, radiotherapy, and palliative chemotherapy have all been employed.

Most authors [10,11,12] claim that BSCC must be considered as an aggressive variant of BCC with a great tendency to both local recurrence and to lymph node and distant metastases. It has been estimated that local recurrence ranges from 12.1% [13] to 45.7% [14] after standard wide surgical excision. In some studies on BSCC not affecting the breast, Mohs micrographic surgery compared to wide surgical excision seems to have a better outcome, since it is linked to lower recurrence rates [15,16]. Leibovich et al., in a series of 98 patients [15], reported a recurrence rate of 4.1% in a 5 year follow-up. In a series of 89 patients with BSCC, Wermker et al. [12] reported a recurrence rate of 4.5% after Mohs micrographic surgery.

In a series of 28 patients with BSCC, Martin RC et al. [10] showed that positive surgical margins, lymphatic invasion, and perineural invasion were risk factors for recurrence, whereas the degree of differentiation and the size of the initial lesion were not significant.

Furthermore, several studies have reported that metastatic rate of BSCC ranged from 2.0% to 8.6% [11,12,13,14,15] compared with 0.1% [16] of more common subtypes of BCC. BSCC is significantly predominant in males, and the age at presentation ranges from 25 to 90 years [15].

BSCC, as well as the other NMSC (BCC and SCC), occurs more frequently on sun-exposed areas, such as head and neck, the most common locations being the central part of the face, including nose [17]. On the contrary, trunk and limbs are less frequently affected. Since the NAC is rarely exposed to large quantities of UV radiations, the occurrence of SCC and of BCC at this site is extremely rare. Almost 62 cases of BCC and 14 cases of SCC have, so far, been reported. Furthermore, BCC and SCC of the NAC have been considered more aggressive, with a high tendency to lymphatic spread. In a series of 34 cases of BCC of the NAC, Ferguson et al. [18] reported a 9.1% incidence of axillary metastases. The greater potential for metastases in this location is likely due to the rich lymphatic and capillary flows in the NAC, that may provide a direct route for tumor spread. In relation to UV exposure, according to the American Cancer Society [19], there are many less common sources of UV rays other than sun exposure, such as: Phototherapy (UV therapy), Black-light lamps, Mercury-vapor lamps, High-pressure xenon and xenon-mercury arc lamps, plasma torches, and welding arcs. Our patient was a retired bank employee, and, therefore, was not at risk for prolonged exposure to the sun; furthermore, he had never been exposed to any of the above listed unusual UV sources. 

Several studies have shown that, in patients with breast cancer, the proximity to the nipple is a risk factor for axillary lymph node involvement [20,21,22]. In one of these studies [20], the incidence of axillary lymph node metastases was significantly higher in patients with nipple involvement (36.71%) than in patients with unifocal tumors (9.76%). BCC and SCC with no evidence of metastases were treated by different approaches, such as wide surgical excision, Mohs micrographic surgery, cryosurgery, electrodessication, and photodynamic therapy [18,23,24]. However, some authors suggest a questionable more aggressive adjuvant approach to BCC and SCC of the NAC, such as mastectomy (with or without lymphadenectomy), and radiotherapy [25,26,27,28,29,30]. We disagree with such an aggressive approach, and, on the contrary, we favor less invasive strategies, regardless of the onset and of the size of the lesion, when radiological findings are normal.

To the best of our knowledge, the case we present is the first ever reported case of BSCC on the NAC. The clinical differential diagnosis of BSCC of the NAC mainly included Paget’s disease, Bowen’s disease, Sebaceous Gland carcinoma, BCC and SCC lesions. Breast ultrasound, mammogram, MRI were all performed to rule out any mammary involvement. The definitive diagnosis of BSCC was made only after punch biopsy and microscopic examination. Finally, histological evidence of BSCC, CT scan negative for metastases, the presence of high-risk factors such as gender (male), size (≥20 mm) and site, lead us to perform a simple mastectomy with sentinel lymph node biopsy. Intraoperative histological examination did not show nodal metastases, so lymphadenectomy was not required. This is the first case of BSCC of the NAC, and certainly we cannot give any clinical general advice on the best therapeutic strategy, as this cannot be based solely on a single case report. In our paper, we only present our therapeutic approach that was effective and without recurrence or metastases after 18 months. Therefore, our strategy, together with the others, should be considered for future cases, and, in any case, further studies on larger cohorts should be advocated. There is still a lack of consensus on definition, histological features, and treatment of BSCC, which likely leads to misdiagnosis and inappropriate management of the disease. Incisional biopsy with immunohistochemical studies is mandatory for a definitive diagnosis. Simple mastectomy with sentinel lymph node biopsy is an effective and safe option for treatment of BSCC on NAC.

## 4. Conclusions

To the best of our knowledge, this is the first case of BSCC of the NAC reported in the literature. BSCC is an aggressive neoplasm with a propensity for local recurrence and potential for distant metastatic spread.

## Data Availability

Not applicable.

## Supplementary Materials

The following supporting information can be downloaded at: https://www.mdpi.com/article/10.3390/medicina59020316/s1, Care Checklist.
